# Influence of Intravenous Magnesium Sulfate Infusion on the Subjective Postoperative Quality of Recovery: A Meta-Analysis of Randomized Controlled Trials

**DOI:** 10.3390/nu16142375

**Published:** 2024-07-22

**Authors:** Kuo-Chuan Hung, Li-Chen Chang, Chun-Ning Ho, Chih-Wei Hsu, Jheng-Yan Wu, Yao-Tsung Lin, I-Wen Chen

**Affiliations:** 1 Department of Anesthesiology, Chi Mei Medical Center, Tainan City 71004, Taiwan; ed102605@gmail.com (K.-C.H.);; 2 Department of Anesthesiology, E-Da Hospital, I-Shou University, Kaohsiung City 82445, Taiwan; 3 School of Medicine, College of Medicine, National Sun Yat-sen University, Kaohsiung 80424, Taiwan; 4 Department of Psychiatry, Kaohsiung Chang Gung Memorial Hospital and Chang Gung University College of Medicine, Kaohsiung City 83301, Taiwan; 5 Department of Nutrition, Chi Mei Medical Center, Tainan City 71004, Taiwan; 6 Department of Anesthesiology, Chi Mei Medical Center, Liouying, Tainan City 73657, Taiwan

**Keywords:** quality of recovery, magnesium sulfate, general anesthesia, meta-analysis, postoperative

## Abstract

This meta-analysis investigated the effects of intravenous magnesium sulfate on the postoperative recovery quality, as assessed using the Quality of Recovery (QoR) questionnaire, in adult surgical patients. Seven randomized controlled trials involving 622 patients were included. Compared with the placebo, magnesium sulfate significantly improved the global QoR score on postoperative day 1 (standardized mean difference [SMD]: 1.24; 95% confidence interval: 0.70—1.78; *p* < 0.00001). It also enhanced specific QoR dimensions, with substantial effects on pain (SMD: 1, *p* < 0.00001) and physical comfort (SMD: 0.85, *p* < 0.0001), a moderate effect on emotional state (SMD: 0.65, *p* = 0.002), and small improvements in physical independence (SMD: 0.43, *p* < 0.00001) and psychological support (SMD: 0.37, *p* < 0.0001). In addition, magnesium sulfate reduced the intraoperative opioid consumption (SMD: −0.66, *p* < 0.0001), postoperative pain severity, and the incidence of postoperative nausea and vomiting (risk ratio: 0.48, *p* = 0.008). The extubation times were unaffected, whereas the post-anesthesia care unit stay was slightly prolonged. These findings highlight the potential of magnesium sulfate as a valuable adjunct for multimodal analgesia and enhanced recovery. Future studies should aim to elucidate the optimal dosing strategies, timing of administration, and specific surgical populations that may derive maximum benefits.

## 1. Introduction

Postoperative recovery quality has become an increasingly important consideration in surgical care owing to its substantial impact on patients’ well-being and satisfaction [1,2]. In the past, objective outcome measurements, such as the surgical complications, incidence of postoperative nausea and vomiting (PONV), and duration of hospital stay, were commonly utilized to evaluate the postoperative recovery status [3]. However, with the continuous development of surgical and anesthetic techniques, the subjective recovery quality following surgery has gained considerable attention in clinical settings [4,5,6]. Poor recovery quality after surgery can not only diminish a patient’s quality of life but also be associated with postoperative complications [7,8,9]. Thus, the implementation of effective strategies to improve the subjective recovery quality is crucial for optimizing surgical outcomes and patient care. Quality of Recovery (QoR) questionnaires, such as the QoR-15 and QoR-40, are patient-reported outcome measures that evaluate various aspects of postoperative recovery, such as pain, physical comfort, emotional well-being, and independence [10,11]. These questionnaires have been extensively used to evaluate the effectiveness of interventions designed to enhance the subjective recovery quality [12,13,14,15].

Magnesium is an N-methyl-d-aspartate (NMDA) receptor antagonist [16] that has been explored for its potential to improve postoperative pain outcomes for many years. It is involved in the regulation of pain pathways and inflammatory responses, making it a promising target for the enhancement of postoperative pain outcomes [17,18,19]. Cumulative evidence has indicated that intravenously administered magnesium sulfate may reduce postoperative pain, opioid consumption, and the incidence of postoperative complications (e.g., shivering) [20,21,22,23,24,25]. However, its efficacy in improving the subjective recovery quality following surgery remains to be elucidated. Several randomized controlled trials (RCTs) have investigated the effects of intravenous magnesium sulfate infusion on the quality of postoperative recovery, as measured using a QoR questionnaire [26,27,28]. However, these studies have yielded inconsistent results. While some studies have reported substantial improvements in global QoR scores [26,27], one study found no significant benefits [28]. Our recent meta-analysis revealed that NMDA receptor antagonists such as ketamine or esketamine can improve the subjective recovery quality [29]. To the best of our knowledge, no comprehensive meta-analysis has been conducted to synthesize the available evidence on the efficacy of magnesium sulfate administration in improving the recovery quality after surgery. Therefore, the present meta-analysis aimed to evaluate the effects of magnesium sulfate treatment on the postoperative QoR in adult patients undergoing surgery. By offering a thorough and current synthesis of the available evidence, this meta-analysis seeks to inform clinical decision-making and guide future research in this area.

## 2. Materials and Methods

### 2.1. Data Source and Protocol Registration

This meta-analysis was conducted in accordance with the Preferred Reporting Items for Systematic Reviews and Meta-Analyses guidelines. To maintain transparency and minimize reporting bias, the study protocol (registration number: CRD42024556417) was registered with the International Prospective Register of Systematic Reviews before initiating the search for relevant literature. An extensive search of the relevant literature was conducted to select RCTs that compared the effects of intravenous magnesium sulfate infusion with a placebo or no treatment in terms of the enhancement of the quality of postoperative recovery. We explored several databases, including Embase, Medline, Cochrane Central Register of Controlled Trials, and Google Scholar, from their inception until June 9, 2024. Our search strategy involved the use of Medical Subject Headings and keywords, focusing on terms associated with magnesium sulfate, postoperative recovery quality, and RCTs. A detailed methodology for the searches in one of the databases is presented in Table 1. The bibliographies of the included studies and relevant systematic reviews were also manually reviewed to identify additional trials that were not initially identified. No restrictions were imposed on the language or date of publication. Two independent reviewers conducted the initial screening of titles and abstracts to identify relevant articles. Subsequently, the full texts of these articles were reviewed to confirm their eligibility based on the inclusion criteria. Any discrepancies between reviewers were resolved through discussion or consultation with a third reviewer, if necessary.

### 2.2. Selection Criteria

The criteria for including and excluding studies in this meta-analysis were based on the Population, Intervention, Comparator, Outcomes, Study design (PICOS) framework, considering adults aged over 18 years undergoing surgeries under general anesthesia and excluding pediatric and pregnant patients or those under regional anesthesia. The intervention group focused on the effects of perioperative intravenous magnesium sulfate infusion, whereas the control group consisted of patients receiving either a placebo or no treatment. The primary endpoint was the subjective quality of postoperative recovery that was measured using validated tools such as the QoR-40 and QoR-15 questionnaires. Only RCTs were included, and no restrictions on language or publication status were imposed.

Studies were excluded from the meta-analysis if magnesium sulfate was administered through non-intravenous routes, such as intramuscular, oral, intra-articular, or epidural routes. Studies in which magnesium sulfate was not used intraoperatively, those involving patients undergoing procedures under sedation only, and those in which magnesium sulfate was part of an opioid-free anesthetic technique were also excluded. Any disagreements among the reviewers were resolved through discussions or consultation with a third reviewer, if necessary.

### 2.3. Data Extraction

Data from the included studies were meticulously extracted by two independent reviewers using a standardized form designed to capture comprehensive details. The data included the study characteristics (e.g., first author, sample size), participant demographics (e.g., age, sex, American Society of Anesthesiologists (ASA) physical status), intervention specifics (e.g., magnesium sulfate dosage), and outcome measures. Furthermore, information that was vital for the evaluation of the risk of bias in each study was collected. In cases of missing data, the corresponding authors were contacted via email to retrieve the data. Any discrepancies during data extraction were resolved through discussions between the reviewers or consultation with a third reviewer, if necessary.

### 2.4. Outcomes and Definition

The primary outcome was the global QoR scores assessed on the first postoperative day (POD 1) using validated tools (i.e., QoR-40 and QoR-15 questionnaires). The secondary outcomes included additional QoR dimensions on POD 1, severity of postoperative pain measured in the post-anesthesia care unit (PACU) or at 24 h postoperatively, intraoperative opioid use, extubation times, incidence of PONV, and duration of PACU stay.

### 2.5. Quality Assessment

The quality of the included studies was independently evaluated by two reviewers using the Cochrane Risk of Bias tool (RoB 2), which was specifically designed for RCTs. The RoB 2 assessed several key domains: (1) risk of bias stemming from the randomization process; (2) deviations from the intended interventions; (3) missing outcome data; (4) measurement inaccuracies of the outcome; (5) and selective reporting of results. According to the RoB 2 criteria, each domain was rated as low, some concern, or a high risk of bias. An overall risk of bias rating was applied to each study based on these domain-specific assessments. Any discrepancies between reviewers were resolved through discussion or consultation with a third reviewer, if necessary.

### 2.6. Certainty of Evidence Assessment

The certainty of the evidence for each outcome was independently evaluated by two reviewers using the Grading of Recommendations, Assessment, Development, and Evaluation (GRADE) approach, considering the following five domains: risk of bias, inconsistency, indirectness, imprecision, and publication bias. It was rated as high, moderate, low, or very low based on the GRADE approach. High certainty indicated that further research was extremely unlikely to change our confidence in the effect estimate; moderate certainty indicated that further research could significantly impact our confidence in the estimate and might alter it; low certainty suggested that further research was extremely likely to significantly impact our confidence in the estimate and was expected to change it; and very low certainty implied that the effect estimate was highly uncertain. Any disagreements between the reviewers were resolved through discussion or consultation with a third reviewer, if necessary.

### 2.7. Statistical Analysis

The statistical analysis was conducted using RevMan version 5.4 (The Cochrane Collaboration, Copenhagen, Denmark). For the continuous outcomes, the mean difference (MD) or standardized mean difference (SMD) with a 95% confidence interval (CI) was calculated. The SMD was used when the included studies evaluated the same outcome using different scales or measurement tools. The effect size for the SMD was categorized as follows: 0.2, indicating a small effect; 0.5, a moderate effect; and 0.8, a large effect. For the dichotomous outcomes (e.g., PONV incidence), the risk ratio (RR) with 95% CIs was calculated. The heterogeneity among the included studies was assessed using the chi-square test and quantified using the *I*^2^ statistic. An *I*^2^ value of 25%, 50%, or 75% was considered to indicate low, moderate, or high heterogeneity, respectively. A random-effects model was used for the meta-analysis, regardless of the heterogeneity.

Sensitivity analyses were conducted to evaluate the robustness of the results by excluding the studies one at a time. If 10 or more studies were included in the meta-analysis for a specific outcome, publication bias was assessed using a funnel plot and Egger’s test [30]. Asymmetry in the funnel plot and a *p*-value < 0.1 according to Egger’s test were considered to indicate potential publication bias. Subgroup analyses of the primary outcome were conducted based on the type of QoR scale to identify potential sources of heterogeneity. All the statistical tests were two-tailed, and a *p*-value < 0.05 was considered statistically significant, except for the assessment of heterogeneity and publication bias, where a *p*-value < 0.1 was utilized.

## 3. Results

### 3.1. Selection and Characteristics of Studies

**Figure 1:**
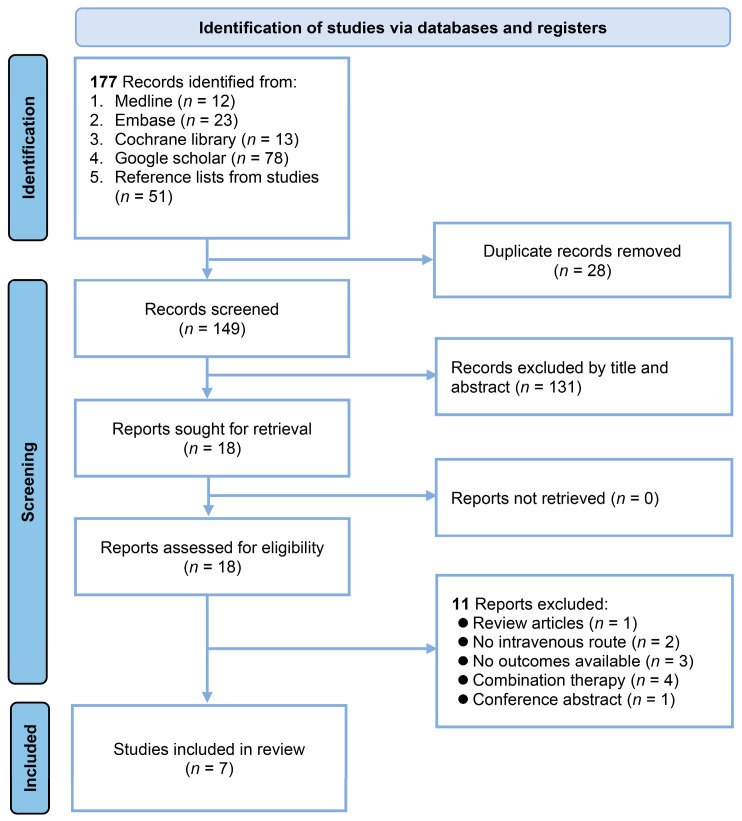
Flowchart of the study selection process.

The initial search of the various databases yielded 177 records (Figure 1). A review of duplicates led to the removal of 28 records, leaving 149 for title and abstract screening. Of these, 131 were deemed unsuitable owing to the failure to meet the inclusion criteria. Further assessment was conducted of 18 studies after full-text retrieval. A comprehensive review of the 18 full-text studies led to the exclusion of 11 studies for the following reasons: review article (*n* = 1), intravenous magnesium sulfate infusion was not employed (n = 2), lacked the outcomes of interest (*n* = 3), magnesium sulfate was used as a combination therapy (*n* = 4), and only a conference abstract (*n* = 1). Ultimately, seven studies [26,27,28,31,32,33,34] were included in the qualitative synthesis and subsequent meta-analysis.

This meta-analysis included seven studies involving 622 patients. The details of these studies are presented in Table 2. The number of participants in the individual studies varied from 46 to 134, with their ages ranging between 18 and 67 years. The proportion of men across the studies ranged from 0% to 53%. Four of the studies provided body mass index data, with values ranging from 24 to 26 kg/m^2^ [26,31,33,34]. Two of the studies reported only patient weights (range: 55–58 kg) [27,28], whereas one study did not provide relevant demographic data [32]. Most studies included patients classified as ASA physical status I–II. However, one study included patients categorized as ASA physical status II–III [34]. Although the studies covered various surgical types, they predominantly involved short-duration procedures, with surgical times ranging from 35 min to approximately 105 min. Intravenous magnesium sulfate infusion was employed intraoperatively for the intervention, with the bolus dosages varying from 20 to 50 mg/kg. In all the studies, normal saline was used in the control groups. The postoperative recovery quality was assessed using either the QoR-40 (five trials) [26,27,28,31,32] or QoR-15 (two trials) [33,34] questionnaire. The studies included in this meta-analysis were conducted across a diverse set of global locations, including the USA, China, Turkey, and Korea. 

### 3.2. Quality of Studies

**Figure 2:**
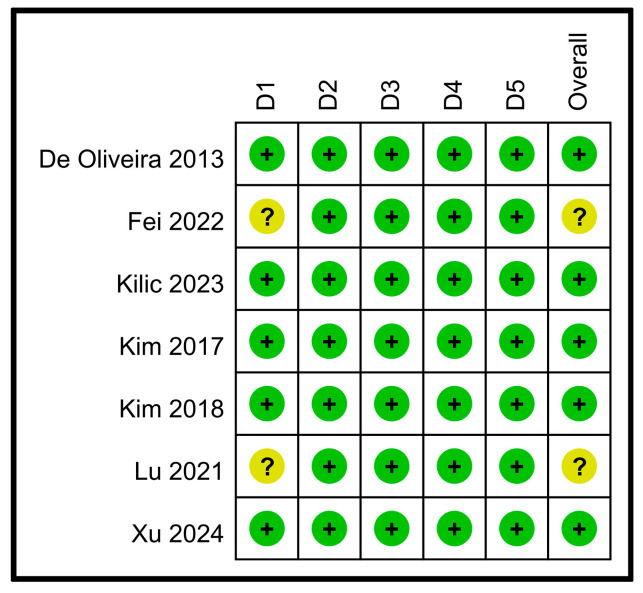
Risk of bias assessment [26,27,28,31,32,33,34]. D1: bias arising from the randomization process; D2: bias due to deviations from the intended interventions; D3: bias due to missing outcome data; D4: bias in the measurement of the outcome; and D5: bias in the selection of the reported results. Green with Plus Sign (+): Indicates a low risk of bias; Yellow with Question Mark (?): Indicates some concerns regarding the risk of bias.

Two studies lacked explicit details regarding the method of sequence generation or allocation concealment, potentially introducing selection bias [31,33]. The risk of bias for these two studies [31,33] was considered to be a concern. The other studies were considered to have a low risk of bias across all the domains. In terms of the overall risk of bias, two studies [31,33] were considered to have some concern, whereas five were considered to have a low risk of bias (Figure 2).

### 3.3. Outcomes 

#### 3.3.1. Primary Outcomes—Global QoR Scores on POD 1

**Figure 3:**
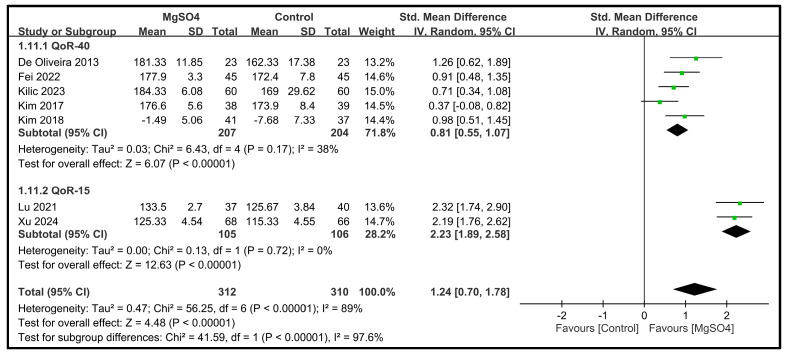
Forest plot showing the impact of magnesium sulfate (MgSO4) on the quality of recovery (QoR) on postoperative day (POD) 1 [26,27,28,31,32,33,34]. IV: invariance, Std: standardized, CI: confidence interval.

Seven studies involving 622 patients reported data on the global QoR scores on POD 1. The study by Kim et al. [27] only reported the difference in the global QoR scores, whereas the other studies presented endpoint values. The pooled results indicated that intravenous magnesium sulfate infusion significantly improved the global QoR scores on POD 1 compared to the placebo (SMD: 1.24; 95% CI: 0.70 to 1.78; *p* < 0.00001) [26,27,28,31,32,33,34] (Figure 3). However, significant heterogeneity was observed among the studies (I^2^ = 89%). Subgroup analysis based on the type of QoR scale revealed that the beneficial effect of magnesium sulfate on the global QoR scores on POD 1 was consistent across the different QoR scales. Moreover, the heterogeneity was not significant in each subgroup, suggesting that the heterogeneity was attributed to the type of QoR scale used.

#### 3.3.2. Secondary Outcomes—QoR Dimensions

**Figure 4:**
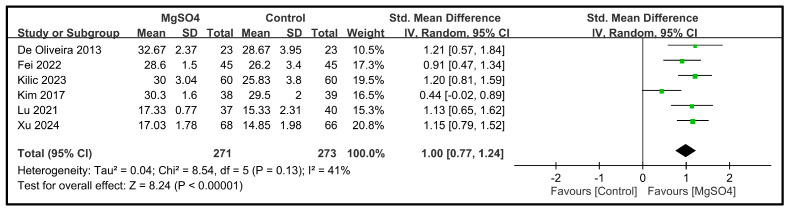
Forest plot showing the impact of magnesium sulfate (MgSO4) on the pain dimension on postoperative day (POD) 1 [26,28,31,32,33,34]. IV: invariance, Std: standardized, CI: confidence interval.

**Figure 5:**
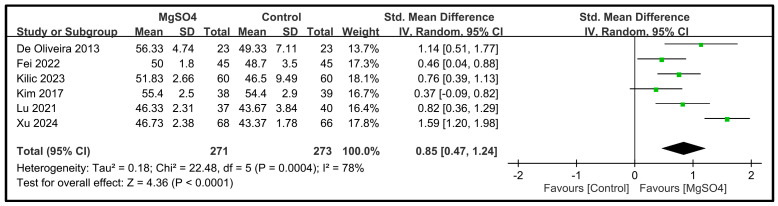
Forest plot showing the impact of magnesium sulfate (MgSO4) on the physical comfort dimension on postoperative day (POD) 1 [26,28,31,32,33,34]. IV: invariance, Std: standardized, CI: confidence interval.

**Figure 6:**
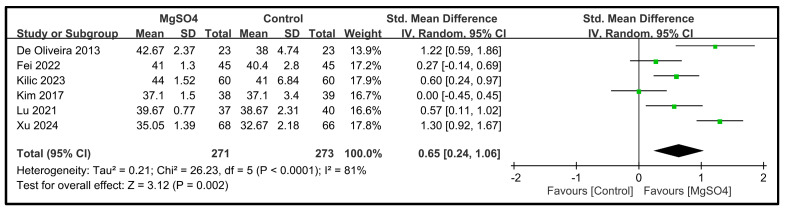
Forest plot showing the impact of magnesium sulfate (MgSO4) on the emotional dimension on postoperative day (POD) 1 [26,28,31,32,33,34]. IV: invariance, Std: standardized, CI: confidence interval.

**Figure 7:**
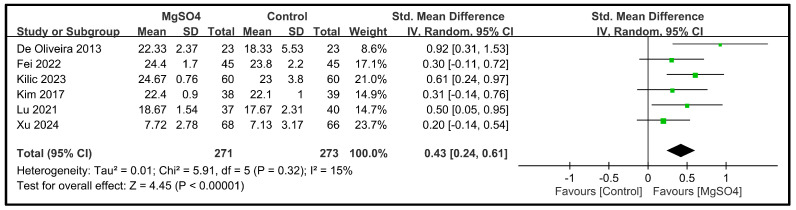
Forest plot showing the impact of magnesium sulfate (MgSO4) on the physical independence dimension on postoperative day (POD) 1 [26,28,31,32,33,34]. IV: invariance, Std: standardized, CI: confidence interval.

**Figure 8:**
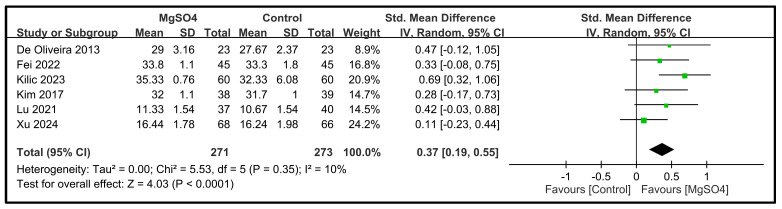
Forest plot showing the impact of magnesium sulfate (MgSO4) on the psychological support dimension on postoperative day (POD) 1 [26,28,31,32,33,34]. IV: invariance, Std: standardized, CI: confidence interval.

Six studies involving a total of 544 patients reported data on the five dimensions of the QoR scores on POD 1 [26,28,31,32,33,34]. The pooled results indicated that magnesium sulfate administration significantly improved the pain dimension (SMD: 1.00; 95% CI: 0.77 to 1.24; *p* < 0.00001, *I*^2^ = 41%) (Figure 4), physical comfort (SMD: 0.85; 95% CI: 0.47 to 1.24; *p* < 0.0001, *I*^2^ = 78%) (Figure 5), emotional state (SMD: 0.65; 95% CI: 0.24 to 1.06; *p* < 0.002, *I*^2^ = 81%) (Figure 6), physical independence (SMD: 0.43; 95% CI: 0.24 to 0.61; *p* < 0.00001, *I*^2^ = 15%) (Figure 7), and psychological support (SMD: 0.37; 95% CI: 0.19 to 0.55; *p* < 0.0001, *I*^2^ = 10%) (Figure 8) compared with the placebo. In brief, the effect sizes were large for two dimensions (i.e., pain and physical comfort), moderate for one dimension (i.e., emotional state), and small for two dimensions (i.e., physical independence and psychological support). Among the five dimensions analyzed, physical comfort (*I*^2^ = 78%) and emotional state (*I*^2^ = 81%) exhibited significant heterogeneity. The other domains showed no significant heterogeneity.

#### 3.3.3. Secondary Outcome—Intraoperative Opioid Use and Other Recovery Characteristics

**Figure 9:**
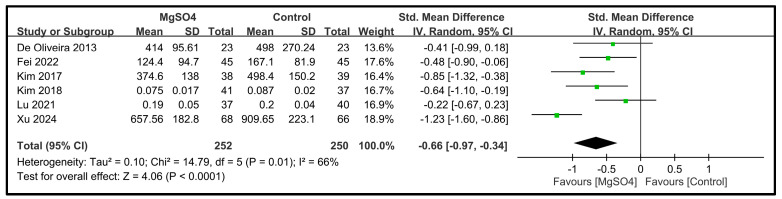
Forest plot showing the impact of magnesium sulfate (MgSO4) on the intraoperative remifentanil use [26,27,28,31,33,34]. IV: invariance, Std: standardized, CI: confidence interval.

Six studies involving 502 patients reported data on intraoperative remifentanil consumption [26,27,28,31,33,34]. The pooled results indicated that magnesium sulfate administration significantly reduced the remifentanil use compared with the placebo (SMD: −0.66; 95% CI: −0.97 to −0.34; *p* < 0.0001) (Figure 9). The effect size of −0.66 suggests a moderate reduction in the intraoperative remifentanil use with magnesium sulfate administration. The forest plot showed that six studies favored magnesium sulfate over the control, with effect estimates ranging from −1.23 to −0.22. Moderate heterogeneity was observed among the studies (*I*^2^ = 66%), indicating some variability in the effect estimates across the studies.

**Figure 10:**
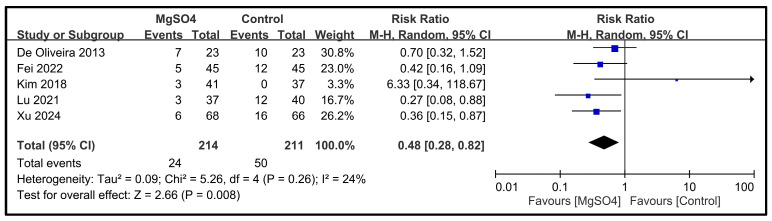
Forest plot showing the impact of magnesium sulfate (MgSO4) on the incidence of postoperative nausea and vomiting [26,27,31,33,34]. CI: confidence interval.

The effect of magnesium sulfate on PONV was evaluated across five studies involving a total of 425 participants [26,27,31,33,34]. Collectively, these studies reported 24 and 50 events in the magnesium and control groups, respectively. The pooled RR was 0.48, suggesting that the administration of magnesium sulfate was associated with a significantly reduced risk of PONV compared with the administration of the control (95% CI: 0.28 to 0.82; *p* = 0.008) (Figure 10). The heterogeneity among the included studies was relatively low (*I*^2^ = 24%), indicating minor variations in the effect sizes reported by the different studies. The results of the individual studies varied, with most studies showing a protective effect. However, one study [27] reported a markedly higher RR (i.e., 6.33), suggesting variability in the effect, possibly due to different study conditions or patient populations. 

**Figure 11:**
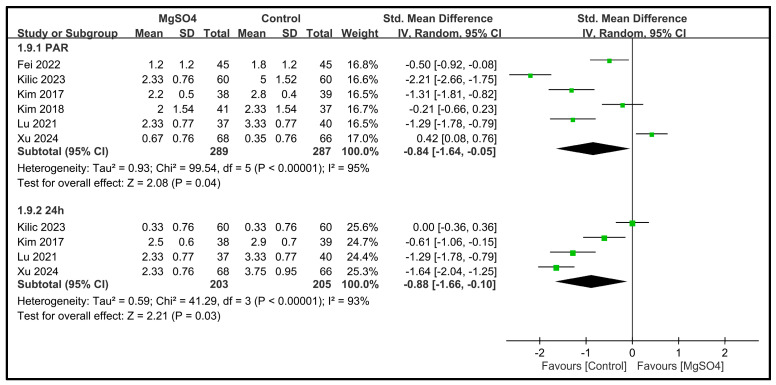
Forest plot showing the impact of magnesium sulfate (MgSO4) on the severity of pain immediately after surgery (i.e., post-anesthesia care unit) [27,28,31,32,33,34] or at 24 h postoperatively [28,32,33,34]. CI: confidence interval. IV: invariance, Std: standardized, CI: confidence interval.

Six studies involving 576 patients were included for assessment of the pain severity in the PACU [27,28,31,32,33,34]. The pooled estimated SMD was −0.84 (95% CI: −1.64 to −0.05, *p* = 0.04) (Figure 11), indicating that the patients receiving magnesium sulfate had significantly lower pain severity in the PACU than the controls. Significant heterogeneity was observed across the studies (*I*^2^ = 95%). For the pain severity at 24 h postoperatively, four studies involving a total of 408 patients were included [28,32,33,34]. The pooled estimated SMD was −0.88 (95% CI: −1.66 to −0.10, *p* = 0.03) (Figure 11), indicating that the patients receiving magnesium sulfate had significantly lower pain severity at 24 h postoperatively compared with the controls. Again, significant heterogeneity was observed across the studies (*I*^2^ = 93%).

**Figure 12:**
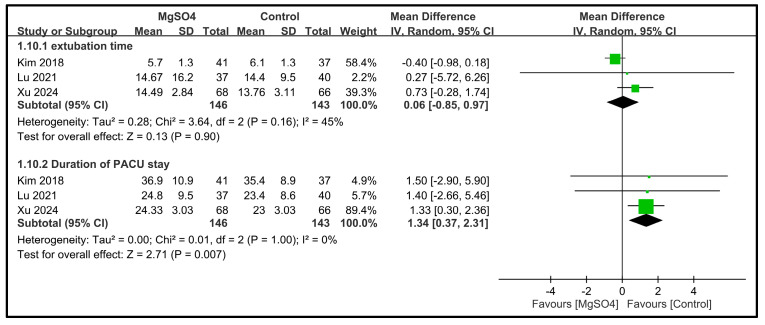
Forest plot showing the impact of magnesium sulfate (MgSO4) on the extubation time [27,33,34] and length of post-anesthesia care unit (PACU) stay [27,33,34]. CI: confidence interval. IV: invariance.

For the extubation time, three studies involving 289 patients were included [27,33,34]. The pooled MD was 0.06 min (95% CI: −0.85 to 0.97) (Figure 12), indicating no significant difference in the extubation time between the magnesium and control groups. No significant heterogeneity was observed across the studies (*I*^2^ = 45%). For the duration of the PACU stay, three studies with 289 patients were included [27,33,34]. The patients receiving magnesium sulfate had a significantly longer PACU stay than the controls (MD: 1.34 min, 95% CI: 0.37 to 2.31, *p* = 0.007) (Figure 12), without heterogeneity. Despite the statistical significance, the clinical relevance of this time difference was minimal.

#### 3.3.4. Sensitivity Analysis

Sensitivity analyses evaluating the robustness of the findings by excluding individual studies revealed consistent results across the studies for the primary outcome (i.e., global QoR scores on POD 1) and all the QoR dimensions, such as pain, physical comfort, emotional state, physical independence, and psychological support (Table 3). Furthermore, the reduction in the intraoperative remifentanil consumption and extubation time was robust in the sensitivity analysis. However, the sensitivity analyses revealed inconsistent results regarding the PONV incidence, postoperative pain scores in the PACU and at 24 h, and duration of PACU stay, suggesting that these outcomes have been influenced by individual studies.

#### 3.3.5. Certainty of Evidence

The GRADE approach was employed to evaluate the certainty of the evidence across the outcomes (Table 3). High certainty was established for the outcomes of the pain dimension, PONV, and extubation time, suggesting that further research is unlikely to change the confidence in the estimated effects. Moderate certainty was observed for the global QoR scores, four QoR dimensions (i.e., physical comfort, emotional state, physical independence, and psychological support), intraoperative remifentanil consumption, and duration of PACU stay, indicating that further studies may potentially impact the effect estimates. Low certainty of evidence was noted for the postoperative pain scores in the PACU and 24 h postoperatively, implying that further research could likely change the confidence in these effect estimates.

## 4. Discussion

The present meta-analysis revealed that intravenous magnesium sulfate infusion during surgery significantly improved the subjective recovery quality on POD 1 compared with a placebo. This beneficial effect was evident across different validated QoR assessment tools (i.e., QoR-40 and QoR-15). Further analysis of the individual QoR dimensions revealed that the administration of magnesium sulfate led to substantial, moderate, and small improvements in pain and physical comfort, emotional state, physical independence, and psychological support, respectively. In addition, magnesium sulfate administration reduced the intraoperative opioid (remifentanil) consumption and PONV incidence. Patients receiving magnesium sulfate also experienced less severe postoperative pain, both in the PACU and 24 h after surgery. While the extubation times were unaffected, a slightly prolonged stay in the PACU was observed with magnesium sulfate administration, although this difference was likely not clinically significant.

Recent studies have highlighted the potential advantages of various perioperative interventions in improving the subjective recovery quality after surgery. For pharmacological interventions, intravenous analgesic adjuvants, such as lidocaine [35] and dexmedetomidine [36], have been demonstrated to enhance the postoperative QoR. For nonpharmacological techniques, the use of nerve blocks in thoracic and breast cancer surgeries has significantly improved the QoR scores while reducing the opioid use and the incidence of nausea and vomiting [37,38]. In addition, transcutaneous electrical acupoint stimulation has been identified as a promising nonpharmacological approach, improving the QoR scores up to 48 h postoperatively and reducing the PONV incidence [39]. The availability of this noninvasive technique could offer a valuable adjunct for enhanced recovery pathways, particularly in patients with contraindications or intolerance to certain medications. Interestingly, the choice of general anesthetic maintenance technique (i.e., intravenous versus inhalational) may also impact the early postoperative QoR, with some evidence suggesting the potential benefits of total intravenous anesthesia using propofol compared to inhalational agents [40,41]. Together, these meta-analyses revealed the efficacy of a wide array of pharmacological and nonpharmacological interventions in enhancing the subjective recovery quality following surgery.

Although two previous meta-analyses had shown that magnesium sulfate can reduce the postoperative opioid requirements or pain score [42,43], the association between magnesium sulfate and recovery quality was not evaluated in these studies. To the best of our knowledge, this meta-analysis is the first to comprehensively evaluate the impact of magnesium sulfate administration on the subjective recovery quality following surgery. Our results indicated a significant improvement in the global QoR scores on POD 1 with magnesium sulfate administration compared with the controls. Essentially, this beneficial effect was consistently observed across the QoR scales (QoR-40 and QoR-15), indicating the robustness of the findings. Additional analyses revealed that magnesium sulfate also enhanced the QoR dimensions, with large improvements in pain and physical comfort, moderate improvement in emotional state, and small but significant improvements in physical independence and psychological support on POD 1. By favorably influencing various aspects of recovery, from physical well-being to emotional and functional status, magnesium sulfate could serve as a valuable adjunct in multimodal analgesic regimens. Its opioid-sparing effects, combined with improvements in patient-reported recovery quality, highlight the potential of magnesium sulfate to facilitate accelerated postoperative convalescence and achievement of discharge criteria.

In addition to improving the pain dimension, intravenous magnesium sulfate infusion enhanced other dimensions, such as physical comfort, emotional state, physical independence, and psychological support. These findings are consistent with our recent publication, which revealed that ketamine and esketamine, which are also NMDA antagonists, can improve the global QoR scores and dimensions [29]. Notably, we observed that magnesium sulfate treatment exerted a considerable effect on pain (SMD: 1) and physical comfort (SMD: 0.85). However, our previous study found that ketamine/esketamine had only a small to moderate effect across these five dimensions, with a range of SMDs between 0.27 and 0.55 on POD 1 [29]. These findings suggest that different NMDA receptor antagonists may have varying effects on the QoR dimensions. Based on these findings, magnesium sulfate administration could be a valuable addition to perioperative care strategies, particularly for enhancing the pain dimensions and patient comfort following surgery. Further research should explore the specific mechanisms through which magnesium sulfate influences these recovery dimensions and establish protocols for its optimal use in clinical settings.

Our findings regarding the postoperative pain scores are consistent with a recent meta-analysis revealing that the administration of magnesium sulfate was associated with lower pain scores up to 24 h postoperatively compared with a placebo [21]. Another novel finding of our study is the reduction in the intraoperative opioid consumption with the use of magnesium sulfate, which has not been reported in previous meta-analyses focusing on the beneficial effects of magnesium sulfate in controlling postoperative pain [42]. This supports the strategy of initiating magnesium sulfate administration either before surgery or immediately after anesthetic induction to maximize its effects. Although we observed the potential of magnesium sulfate administration to reduce the risk of PONV, the robustness of this evidence is limited, as only five studies were included in the analysis of this outcome. This finding is inconsistent with that of a previous meta-analysis [42], which reported that magnesium sulfate did not exert a beneficial effect on PONV. Similarly, our previous study on ketamine/esketamine found that NMDA antagonists did not reduce the risk of PONV [29]. This discrepancy highlights the need for further research to elucidate the role of magnesium sulfate in the management of PONV and to establish more definitive conclusions regarding its efficacy across different postoperative outcomes.

This meta-analysis has several limitations that need to be acknowledged. First, high heterogeneity was observed across certain outcomes, including the global QoR scores on POD 1 and postoperative pain scores. This heterogeneity may have arisen from variations in the surgical procedures, magnesium sulfate dosing regimens, and patient populations across the included studies. Although subgroup analyses were conducted to identify potential sources of heterogeneity, residual heterogeneity could not be fully accounted for. Second, the included studies predominantly involved short-duration surgeries, which potentially limited the generalizability of the findings to more extensive or complex operations. The impact of magnesium sulfate infusion on postoperative recovery after prolonged surgery remains unclear. Third, although the meta-analysis focused on the subjective recovery quality as the primary outcome, objective measures of functional recovery such as the return to normal activities were not assessed. Evaluation of the effects of magnesium sulfate on these objective outcomes could provide a more comprehensive assessment of postoperative recovery. Fourth, although neuraxial magnesium sulfate was also reported to improve postoperative analgesia [44], we only evaluated the beneficial effect of magnesium sulfate administration on the QoR scores. Finally, the long-term effects of magnesium sulfate infusion, including its impact on chronic postsurgical pain, persistent functional impairment, and healthcare resource utilization, were not explored in this meta-analysis because of the lack of data in the included studies. Despite these limitations, the present meta-analysis provides valuable insights into the potential benefits of magnesium sulfate treatment in enhancing the postoperative recovery quality. Future well-designed studies addressing these limitations could further strengthen the evidence base and refine the clinical application of this intervention.

## 5. Conclusions

This meta-analysis provides initial evidence that intravenous magnesium sulfate infusion significantly enhances the subjective recovery quality in the early postoperative period. Furthermore, the administration of magnesium sulfate reduces the intraoperative opioid use, decreases the pain severity within the first 24 h postoperatively, and reduces the incidence of PONV while exerting a minimal effect on the length of stay in the PACU. These findings highlight the potential utility of magnesium sulfate as a valuable adjunct in multimodal analgesia and enhanced recovery. Future studies should aim to further elucidate the optimal dosing strategies, timing of administration, and specific surgical populations that may derive maximum benefits from magnesium sulfate infusion.

## Figures and Tables

**Table 1:** Search strategy for Medline.

1	(“(operative or surgical) adj4 (procedure* or technique*)” or “surger*” or “operation*”).mp.
2	exp “Surgical Procedures, Operative”/
3	(“Magnesium Hydroxide” or “Magnesium Chloride” or “Magnesium Sulfate” or “Magnesium” or “Magnesium Oxide” or “Magnesium Silicates” or “MgSO4”).mp.
4	exp “Magnesium Sulfate”/
5	(“quality of recovery score” or “QoR-40” or “Quality of Recovery-40” or “Quality of Recovery-15” or “QoR-15” or “Quality of Recovery scale” or “quality of recovery”).mp.
6	(1 or 2) and (3 or 4) and 5
7	6 and (((randomized controlled trial or controlled clinical trial).pt. or randomi*ed.ab. or placebo.ab. or drug therapy.fs. or randomly.ab. or trial.ab. or groups.ab.) not (exp animals/ not humans.sh.))

**Table 2:** Characteristics of seven studies with 622 patients.

	Age (Years)	Male (%)	BMI (kg/m^2^)	ASA	N	IV Magnesium Sulfate (Bolus, Infusion)	Control	Procedures	Surgical Duration (Minutes)	QoR	Country
De Oliveira 2013 [26]	53/52	0	24/25	I–II	46	50 mg/kg; 15 mg/kg/h	Saline	Mastectomy	413/442	40	USA
Fei 2022 [31]	44/47	53/47	25/24	I–II	90	20 mg/kg; 20 mg/kg/h	Saline	Airway surgery	38/35	40	China
Kilic 2023 [32]	18–45 ^†^	33/28	na	na	120	30 mg/kg; 9 mg/kg/h	Saline	Septorhinoplasty surgery	na	40	Turkey
Kim 2018 [27]	44/45	0	58/56	I–II	78	20 mg/kg; 20 mg/kg/h	Saline	Thyroidectomy	94/91	40	Korea
Kim 2017 [28]	48/49	0	56/55	I–II	77	20 mg/kg; 20 mg/kg/h	Saline	Breast cancer surgery	105/98	40	Korea
Lu 2021 [33]	47/45	22/28	24/24	I–II	77	20 mg/kg; 20 mg/kg/h	Saline	Cholecystectomy	58/59	15	China
Xu 2024 [34]	67/66	21/24	25/26	II–III	134	40 mg/kg; 15 mg/kg/h	Saline	Total knee replacement	69/71	15	China

^†^ range; na: not available; BMI: body mass index; ASA: American Society of Anesthesiologists physical status classification system; IV Mg: intravenous magnesium sulfate; QoR: Quality of Recovery.

**Table 3:** Summary of the outcomes and certainty of the evidence based on the Grading of Recommendations Assessment, Development and Evaluation (GRADE) approach.

Outcomes	N ^†^	Participants	Certainty Assessment (Domains)					Effect Size [95% CI]	I^2^	Sensitivity Analysis	Certainty
			A	B	C	D	E				
Global QoR scores	7	622	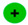	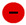	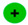	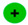	-	SMD 1.24 [0.7, 1.78] *p* < 0.00001	89%	Consistent	⨁⨁⨁◯ Moderate
Pain domain	6	544	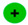	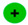	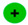	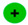	-	SMD 1.0 [0.77, 1.24] *p* < 0.00001	41%	Consistent	⨁⨁⨁⨁ High
Physical comfort	6	544	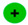	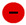	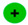	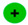	-	SMD 0.85 [0.47, 1.24] *p* < 0.0001	78%	Consistent	⨁⨁⨁◯ Moderate
Emotional domain	6	544	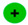	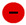	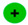	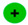	-	SMD 0.65 [0.24, 1.06] *p* = 0.002	81%	Consistent	⨁⨁⨁◯ Moderate
Physical independence	6	544	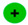	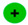	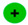	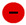	-	SMD 0.43 [0.24, 0.61] *p* < 0.00001	15%	Consistent	⨁⨁⨁◯ Moderate
Psychological support	6	544	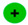	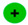	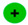	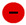	-	SMD 0.37 [0.19, 0.55] *p* < 0.0001	10%	Consistent	⨁⨁⨁◯ Moderate
PONV	5	425	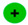	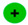	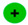	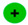	-	RR 0.48 [0.28, 0.82] *p* = 0.008	24%	Inconsistent	⨁⨁⨁⨁ High
Remifentanil	6	502	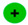	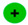	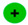	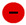	-	SMD −0.66 [−0.97, −0.34] *p* < 0.0001	66%	Consistent	⨁⨁⨁◯ Moderate
Pain score at PACU	6	576	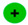	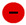	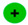	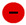	-	SMD −0.84 [−1.64, −0.05] *p* = 0.04	95%	Inconsistent	⨁⨁◯◯ Low
Pain score at 24 h	4	408	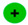	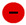	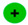	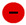	-	SMD −0.88 [−1.66, −0.10] *p* = 0.03	93%	Inconsistent	⨁⨁◯◯ Low
Extubation time	3	289	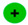	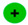	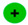	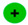	-	MD 0.06 [−0.85, 0.97] *p* = 0.9	45%	Consistent	⨁⨁⨁⨁ High
Duration of PACU stay	3	289	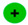	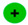	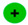	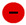	-	MD 1.34 [0.37, 2.31] *p* = 0.007	0%	Inconsistent	⨁⨁⨁◯ Moderate

A: risk of bias; B: inconsistency; C: indirectness; D: imprecision; E: publication bias; green circular icon: not serious; red circular icon: serious. RR: risk ratio; SMD: standardized mean difference; MD: mean difference; CI: confidence interval; QoR: Quality of Recovery; PACU: post-anesthesia care unit; PONV: postoperative nausea and vomiting; publication bias assessed when more than ten studies or datasets were available for a given outcome; ^†^ number of studies or datasets.

## Data Availability

The original contributions presented in this study are included in this article; further inquiries can be directed to the corresponding authors.
